# Extracellular Vesicles Derived from Human Umbilical Cord Mesenchymal Stem Cells Promote Trophoblast Cell Proliferation and Migration by Targeting TFPI2 in Preeclampsia

**DOI:** 10.1155/2023/7927747

**Published:** 2023-08-01

**Authors:** Ying Chen, Chenchen Zhou, Xiaobo Zhao, Ronghua Che, Yuelin Wu, Sheng Wan, Jinda Pei, Liping Yao, Xiaolin Hua

**Affiliations:** ^1^Department of Obstetrics, Shanghai First Maternity and Infant Hospital, Tongji University School of Medicine, Shanghai 201204, China; ^2^Shanghai Key Laboratory of Maternal Medicine, Shanghai First Maternity and Infant Hospital, Tongji University School of Medicine, Shanghai 201204, China; ^3^Department of Ultrasound, Shanghai First Maternity and Infant Hospital, Tongji University school of Medicine, Shanghai 201204, China

## Abstract

Preeclampsia is a pregnancy disorder characterized by systemic organ damage and high blood pressure. It has been reported that microRNA-195 (miR-195) is associated with preeclampsia. In this study, we discovered the target of miR-195 in regulating human extravillous cytotrophoblast-derived transformed cell proliferation and migration. We analyzed the clinicopathological factors of preeclampsia and normal pregnancies. The messenger ribonucleic acid (mRNA) levels of miR-195 and tissue factor pathway inhibitor 2 (TFPI2) were measured in placental tissues derived from normal and preeclampsia patients by real-time polymerase chain reaction (PCR). Human umbilical cord mesenchymal stem cell (hUC-MSC)-derived extracellular vesicles were verified by western blot. HTR8-S/Vneo cell proliferation was evaluated by 3-(4,5-dimethylthiazol-2-yl)-2,5-diphenyltetrazolium bromide, and cell migration rate was assessed by the transwell assay. Relative luciferase activities were measured in TFPI2 wild-type (WT) and mutant cells. miR-195 expression was negatively correlated with TFPI2 mRNA levels in preeclampsia patients. Extracellular vesicles derived from hUC-MSCs enhanced HTR8-S/Vneo cell proliferation and migration. In addition, miR-195 isolated from hUC-MSCs enhanced HTR8-S/Vneo cell proliferation and migration by targeting TFPI2. Our findings demonstrate that the upregulation of miR-195 in extracellular vesicles derived from hUC-MSCs promotes HTR8-S/Vneo cell proliferation and migration by targeting TFPI2.

## 1. Introduction

Preeclampsia is a disorder characterized by a significant amount of proteins in the urine and the occurrence of hypertension. It usually occurs in around 2%–8% of pregnancies [1–3] and is correlated with high morbidity and mortality during pregnancies, with over 50,000 deaths reported worldwide. The most high-risk factors for preeclampsia include diabetes mellitus, overweight, and vascular and connective tissue disorders such as antiphospholipid antibodies and systemic lupus erythematosus [4]. Currently, several therapies such as magnesium sulfate, aspirin, calcium, endothelin antagonists, antioxidants, and cell therapy are available for preeclampsia treatment [5–8]. However, the lack of biomarkers and clinical markers makes it difficult to classify the severity of preeclampsia.

Several studies have reported on the regulatory function of exosomes on trophoblast cell proliferation and migration. For example, exosomal encapsulation of microRNA (miR-125a-5p) was reported to affect HTR8-S/Vneo cell proliferation and migration by regulating vascular endothelial growth factor A [9]. Another study reported that mesenchymal stem cell (MSC)-derived exosomes boosted trophoblast cell proliferation, migration, and invasion by restricting serum glucocorticoid regulated kinase 1 [10]. Additionally, exosomes have been identified as novel biomarkers in multiple diseases [11]. For instance, exosomes derived from umbilical cord MSCs showed a protective effect against chemotherapy-induced apoptosis and ovarian granulosa cell stress [12].

Evidence has shown that miRNAs are abundant in exosomes and can be transported in cell plasma and delivered to recipient cells by exosomes [13]. Given the transport ability of exosomes and the role of miRNAs in various biological processes, exosomes are gaining increasing research attention as a novel biomarker. For instance, miR-195 has been reported to associate with preeclampsia, and overexpression of miR-195 in exosomes alleviates hypoxia-induced damage of trophoblast cells [14]. In this study, we hypothesized that human umbilical cord MSC (hUC-MSC)-derived extracellular vesicles (EVs) could transfer microRNAs and exhibit protective effects against preeclampsia.

Tissue factor pathway inhibitor-2 (TFPI-2) was originally isolated from placental tissue as a glycoprotein. It is a Kunitz-type proteinase inhibitor that inhibits the activation of several metalloproteinases and plays a major role during placenta growth by regulating trophoblast invasion and differentiation [15]. The expression of TFPI-2 has been reported to be significantly upregulated in both the serum and placenta of preeclampsia patients, and TFPI-2 has been demonstrated to play a crucial role in regulating trophoblast cells [16].

## 2. Methods

### 2.1. Clinical Samples

Seventy-four clinical placental tissues, including 34 from preeclampsia patients and 40 from normal control pregnancies, were resected during surgery at Shanghai First Maternity and Infant Hospital, Tongji University School of Medicine. The clinical information for all 74 specimens is presented in Table 1. Preeclampsia was defined in accordance with previous descriptions [17], and patients were excluded based on previously established criteria [18]. The Ethical Committee of Shanghai First Maternity and Infant Hospital, Tongji University School of Medicine, approved this study.

### 2.2. Cell Culture and Transfection

The human extravillous cytotrophoblast-derived transformed cell line, HTR8-S/Vneo, was obtained from Hibio Bio-tech Co., Ltd. (Hangzhou, Zhejiang, China). The HTR8-S/Vneo cells were cultured in Dulbecco's Modified Eagle Medium (DMEM)/Ham's F-12, including 1% nonessential amino acids, 2 m mol/L glutamine, and 10% heat-inactivated fetal bovine serum (all from Invitrogen, Waltham, MA) in an incubator at 37°C in a humidified atmosphere consisting of 5% CO_2_. To explore the role of TFPI2 in HTR8-S/Vneo cell proliferation and migration, TFPI2 small interfering RNA (siRNA) or plasmid was transfected into HTR8-S/Vneo cells. In brief, cells were cultured in 6-well plates and then transfected with siTFPI2 (10 *μ*L), siCon (10 *μ*L), TFPI2 plasmid (4 *μ*g), or plasmid vectors (4 *μ*g) with 10 *μ*L Lipofectamine 2,000 (Invitrogen). Cells were harvested after 48-hr transfection for following experiments. The TFPI2 siRNA was purchased from Invitrogen with the following sequences: sense 5′-AGCCCAUACAAGUAGCUUCAUCUGG-3′, antisense 5′-CCAGAU GAAGCUACUUGUAUGGGCU-3′. The control siRNA sequence: sense 5′-UUCUCCGAACGUGUCACGUTT-3′, antisense 5′-ACGUGACACGUUCGGAGAATT-3′. The TFPI2 plasmid (2,444 bp, NM_006528.3, synthesized by Realgene, Nanjing, China) was subcloned into the pcDNA3.1(+) vector (Invitrogen).

### 2.3. hUC-MSCs Isolation and Identification

The umbilical cords of healthy newborns were obtained and cut into small sections in DMEM medium containing 3% penicillin-streptomycin (Invitrogen). Briefly, tissue separation was performed at the ultraclean workbench. Neonatal umbilical cord tissue was obtained first, then cord blood was separated by squeezing the umbilical cord, and then, the umbilical cord tissue was rinsed in phosphate-buffered saline (PBS) containing 2% penicillin-streptomycin. The umbilical cord tissue after washing was cut into fragments about 1 cm long, and the umbilical vein and umbilical artery were carefully removed. The remaining umbilical tissue was then carefully placed in DMEM medium for further cutting to about 1 mm^3^ fragments. The fragments were collected and placed in a 25 cm^2^ culture flask with 4 mL medium. After 7 days of culture, there were spindle cells around the tissue blocks, namely hUC-MSCs, observed by optical microscope.

### 2.4. Extraction and Identification of EVs

EVs were purified and identified as described previously [10]. hUC-MSCs were cultured without serum for 2 days, and the supernatant was collected and stored at 4°C for 1 week. The supernatant was first centrifuged at 300 × *g* for 10 min and then at 15,000 × g for 20 min. After passing through a 0.22 *μ*m filter, the supernatant was centrifuged at 100,000 × g for 1 hr. The EV suspension was obtained by resuspending the precipitates in PBS. EVs were then identified using a transmission electron microscope. In brief, 15 *μ*L of exosomal suspension was transferred to a copper mesh for a few minutes, and 20 *μ*L of a phosphotungstic acid solution (30 g/L) was added. Samples were then dehydrated under an incandescent lamp for about 10–20 min. Finally, EVs were observed using a transmission electron microscope. The EV suspensions were analyzed using the NanoSight LM 10 instrument (NanoSight Ltd., Amesbury, UK) following the published method [19]. EV markers Alix, cluster of differentiation (CD9), CD81, and CD63 were detected by western blot.

### 2.5. Real-Time Polymerase Chain Reaction

The total RNAs were extracted from placental tissues of preeclampsia patients and their gestational week-matched normal control pregnancies using TRIzol reagent (Invitrogen) [20]. The RNAs were reverse-transcribed into cDNA using Moloney murine leukemia virus reverse transcriptase (Invitrogen). The following primers were used:

  for TFPI2, F: 5′-AGGAAATAACGCGGAGATCTGTCT-3′ and R: 5′-TTAAAATTGCTTCTTCCGAAT-3′;
  for PCNA, F: 5′-TAAAGAAGAGGAGGCGGTAA-3′ and R: 5′-TAAGTGTCCCATGTCAGCAA-3′;
  for Ki67, F: 5′-TCCTTTGGTGGGCACCTAAGACCTG-3′ and R: 5′-TGATGGTTGAGGTCGTTCCTTGATG-3′;
  for GAPDH, F: 5′-GGGAGCCAAAAGGGTCAT-3′ and R: 5′-GAGTCCTTCCACGATACCAA-3′.

The polymerase chain reaction (PCR) assay was performed using a real-time PCR machine (iQ5, BioRad).

### 2.6. Western Blot

Protein levels were measured by western blot, as previously described [21]. EVs or cells were lysed with radioimmunoprecipitation lysis buffer (Invitrogen). In brief, immunoblotting was performed by incubating with corresponding primary antibodies against Alix (ab275377, Abcam, Cambridge, MA), CD63 (ab59479, Abcam), CD9 (ab92726, Abcam), CD81 (ab79559, Abcam), nucleus related antigen 67 (Ki67) (ab16667, Abcam), proliferating cell nuclear antigen (PCNA) (ab29, Abcam), TFPI2 (ab186747, Abcam), and glyceraldehyde-3-phosphate dehydrogenase (GAPDH) (ab8245, Abcam). The membranes were then incubated with secondary antibodies at room temperature for 1 hr and washed with tris-buffered saline and Tween-20 three times. Finally, the membranes were detected using a chemiluminescent detection system.

### 2.7. Luciferase Assay

HEK-293T and HTR8-S/Vneo cells were seeded in 24-well plates prior to transfection. Luciferase reporter plasmids psiCHECK2-TFPI2-WT, psiCHECK2, psiCHECK2-TFPI2-mut, mimic-NC, miR-195 mimics, and pRL-TK plasmid were cotransfected into the cells. After transfection for 48–96 hr, luciferase signals were determined using the Dual Luciferase Assay Kit (Promega, Madison, WI). The primer sequence for TFPI2 3′ untranslated regions (UTR) was 5′-UUAUAUAUAACUAGCUGCUA-3′, while the primer sequence for the mutant 3′ UTR was 5′-UUAUAUAUAACUACGACGAA-3′.

### 2.8. Cell Viability Assay

The 3-(4,5-dimethylthiazol-2-yl)-2,5-diphenyltetrazolium bromide (MTT) assay (Sigma) was used to measure cell viability. HTR8-S/Vneo cells, TFPI2 knockdown, or overexpressed cell lines were seeded at 8 × 10^2^ cells per well in a 96-well plate. After incubation for 2 to 3 days, 50 *μ*g of MTT solution was added to each well and incubated with cells for 4 hr in an incubator. Then, 150 *μ*L of dimethyl sulfoxide was added to each well. The absorbance was detected at 595 nm with a reference wavelength of 650 nm using an ELx-800 University Microplate Reader (BioTek, USA).

### 2.9. Transwell Assay

Transwell assay was used to measure the migration of HTR8-S/Vneo cells under different conditions. In brief, HTR8-S/Vneo cells, TFPI2 knockdown, or overexpressed cell lines (1 × 10^6^ cells/mL) were plated into the Transwell chambers (BD Biosciences, USA) without serum. Meanwhile, the chambers were incubated with medium containing 10% fetal bovine serum for 1 day. After that, cells within the chamber were fixed with PFA, stained with a 0.5% crystal violet solution for half an hour, photographed, and quantified using a standard microplate reader.

### 2.10. Flow Cytometry Analysis

Flow cytometry experiments were performed as previously described [10]. Briefly, hUC-MSCs were stained with anti-CD90 mAb (eBioscience, San Diego, CA), anti-CD105 mAb (eBioscience), anti-CD34 mAb (eBioscience), and anti-CD45 mAb (eNopscoemce) before adding fixation and permeabilization solutions (BD) at passage 3. The flow cytometry results were analyzed using a LSRFortessa flow cytometer and FlowJo software (version 7.6, TreeStar, Ashland, OR).

### 2.11. Statistics

Data were represented as mean ± standard deviation (SD) and analyzed using a two-tailed Student's *t*-test or one-way analysis of variance (ANOVA) with a Tukey post hoc test. Experiments were independently repeated in triplicates. A *p* value < 0.05 was considered statistically significant.

## 3. Result

### 3.1. miR-195 Was Negatively Correlated with mRNA Level of TFPI2 in Preeclampsia Patients

**Figure 1:**
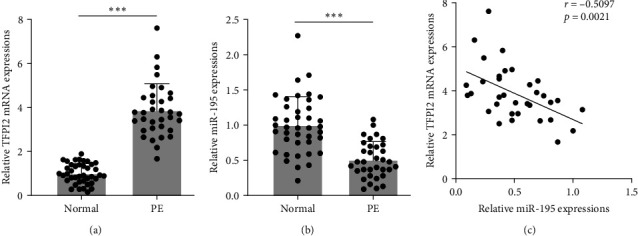
The expression of tissue factor pathway inhibitor 2 (TFPI2) and miR-195 between preeclampsia patients (PE, *n* = 34) and their gestational week-matched normal control pregnancies (normal, *n* = 40). Real-time polymerase chain reaction (RT-PCR) was used to detect the TFPI2 mRNA (a) and miR-195 (b) level in placentas. All data were presented as mean ± SD.  ^*∗∗∗*^*p* < 0.001, Mann–Whitney test. (c) Spearman's rank correlation analysis was employed to analyze the correlations between TFPI2 mRNA and miR-195 in placentas from preeclampsia patients. mRNA, messenger ribonucleic acid; miR-195, microRNA-195.

We first analyzed the clinical characteristics of patients with preeclampsia and normal pregnancies. We found that diastolic blood pressure (DBP), systolic blood pressure (SBP), and proteinuria were significantly higher in preeclampsia patients compared to normal individuals (Table 1). Additionally, the body weight of infants born to preeclampsia patients was lower than that of infants born to the normal group (Table 1). To explore the relationship between TFPI2 and miR-195, we compared the messenger ribonucleic acid (mRNA) expression levels of TFPI2 and miR-195 in the preeclampsia and normal pregnancy cohorts. The results showed a significantly increased TFPI2 mRNA level and decreased expression of miR-195 in preeclampsia patients compared to normal samples (Figures 1(a) and 1(b)). Notably, a significant negative correlation (*p* = 0.0021) was observed between the mRNA level of TFPI2 and miR-195 in preeclampsia patients (Figure 1(c)). These results suggest that TFPI2 might be a potential target of miR-195.

### 3.2. Isolation of EVs from hUC-MSCs

**Figure 2:**
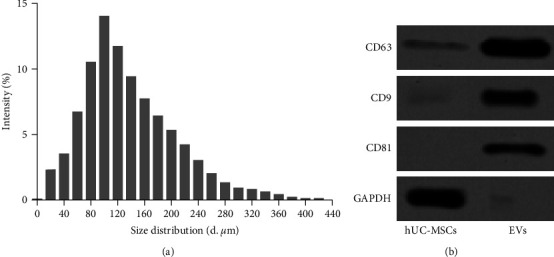
Isolation and identification of human umbilical cord mesenchymal stem cells (hUC-MSCs) and hUC-MSC-derived extracellular vehicles (EVs). (a) Size of the particles in the isolated EV mixture obtained through nanoparticle tracking analysis. (b) CD81, CD9, and CD63 expression detection by western blot.

We next isolated hUC-MSCs and EVs derived from hUC-MSCs. The size of the particles in the isolated EV mixture was analyzed through nanoparticle tracking analysis (Figure 2(a)). In addition, we detected the expression of EV markers. Consistently, high expression of CD63, CD81, and CD9 was observed in EVs but not in hUC-MSC cells (Figure 2(b)). In addition, Alix expression was abundant in EVs, while weak in hUC-MSC cells (Figure S1). The above results indicate that EVs were successfully obtained from hUC-MSCs.

### 3.3. hUC-MSC-Derived EVs Enhanced HTR8-S/Vneo Cell Proliferation and Migration

**Figure 3:**
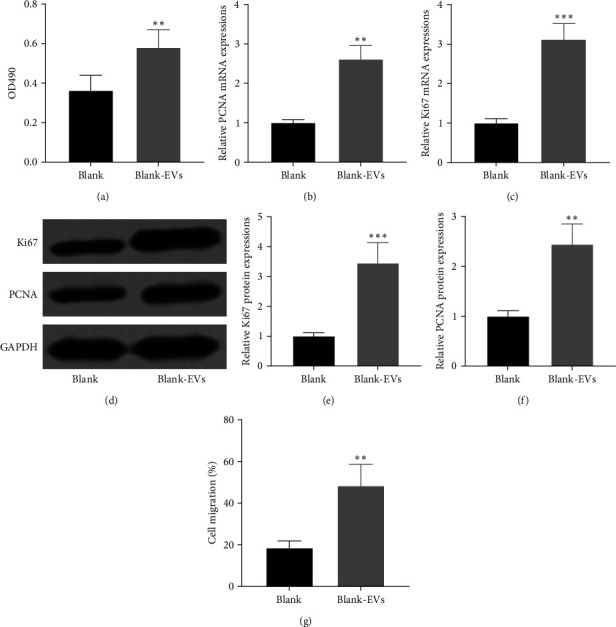
Extracellular vehicles (EVs) derived from human umbilical cord mesenchymal stem cells (hUC-MSCs) elevated HTR8-S/Vneo cell proliferation and migration abilities. (a) HTR8-S/Vneo cell proliferation rate was examined by MTT assay. mRNA levels of PCNA (b) and Ki67 (c) were examined by qRT-PCR. (d) Western blotting was used to measure the protein expressions of PCNA and Ki67. Relative expression was normalized to Blank (e and f). (g) HTR8-S/Vneo cell migration ability by transwell assay (*n* = 3).  ^*∗∗*^*p* < 0.01 and  ^*∗∗∗*^*p* < 0.001 compared to Blank and Mann–Whitney test. Data were presented as mean ± SD. Ki67, nucleus related antigen 67; mRNA, messenger ribonucleic acid; MTT, 3-(4,5-dimethylthiazol-2-yl)-2,5-diphenyltetrazolium bromide; PCNA, proliferating cell nuclear antigen; qRT-PCR, quantitative reverse transcription polymerase chain reaction.

To determine whether hUC-MSC-derived EVs play a role in cell proliferation, we used HTR8-S/Vneo cells, a human extravillous cytotrophoblast-derived transformed cell line, and performed an MTT assay. We compared HTR8-S/Vneo cell proliferation with or without hUC-MSC-derived EVs. The result, from the optical density 490 value, indicated an increased cell proliferation rate when HTR8-S/Vneo cells were cultured with hUC-MSC-derived EVs (defined as blank-EVs), compared to HTR8-S/Vneo cells without hUC-MSC-derived EVs (defined as blank; Figure 3(a)). Consistently, the mRNA and protein levels of two proliferative markers, PCNA and Ki67, were upregulated in cells with hUC-MSCs-derived EVs (Figures 3(b) and 3(f)). Moreover, the transwell assay showed that hUC-MSC-derived EVs also elevated the HTR8-S/Vneo cell migration (Figure 3(g)). In conclusion, hUC-MSC-derived EVs could enhance HTR8-S/Vneo cell proliferation and migration.

### 3.4. hUC-MSC-Derived EVs Regulated the Expression of miR-195 and TFPI2

**Figure 4:**
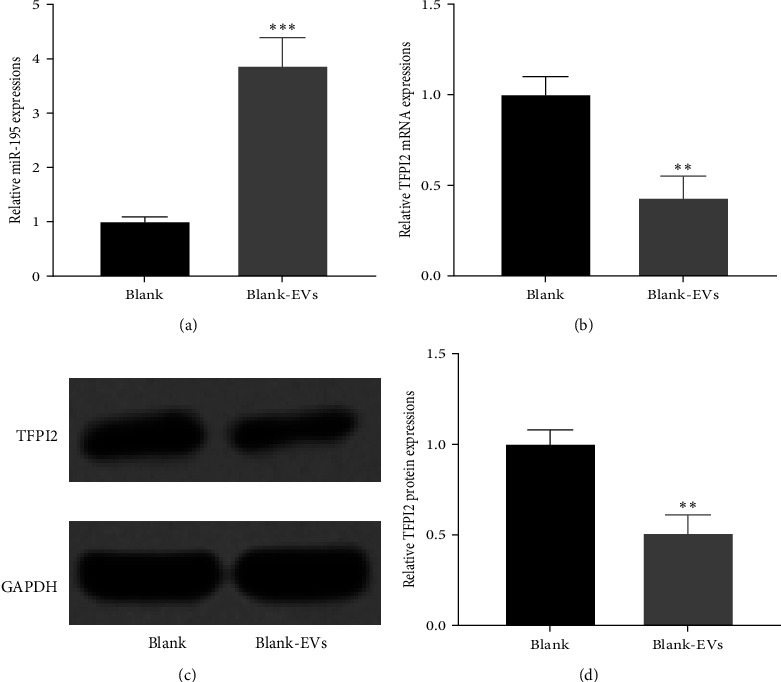
Coculture of human umbilical cord mesenchymal stem cell (hUC-MSC)-derived extracellular vesicles (EVs) with HTR8-S/Vneo cells elevated the expressions of miR-195 and suppressed the expressions of tissue factor pathway inhibitor 2 (TFPI2). qRT-PCR was employed to determine the miR-195 (a) and TFPI2 mRNA (b) levels from cocultured cells. (c) Protein expressions of TFPI2 from the cocultured cells (*n* = 3).  ^*∗∗*^*p* < 0.01 and  ^*∗∗∗*^*p* < 0.001 compared to Blank and Mann–Whitney test. Data were presented as mean ± SD. mRNA, messenger ribonucleic acid; miR-195, microRNA-195; qRT-PCR, quantitative reverse transcription polymerase chain reaction.

To further explore the role of hUC-MSC-derived EVs in regulating TFPI2 and miR-195 expression, we detected the mRNA levels of miR-195 and TFPI2 in HTR8-S/Vneo cells cocultured with hUC-MSC-derived EVs (defined as blank-EVs group) or without EVs (defined as blank group). Compared with the control group, we observed significantly enhanced expression of miR-195, but decreased expression of TFPI2, in the cells cocultured with hUC-MSC-derived EVs (Figures 4(a) and 4(b)). Consistently, the protein level of TFPI2 was also decreased in the presence of EVs (Figures 4(c) and 4(d).

### 3.5. TFPI2 Was Targeted and Downregulated by miR-195

**Figure 5:**
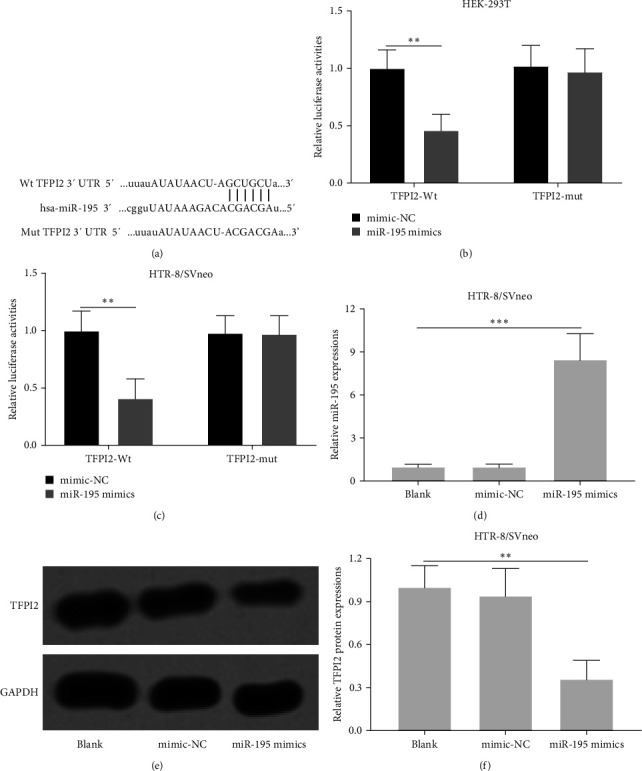
miR-195 targeted tissue factor pathway inhibitor 2 (TFPI2) and regulated the expression of TFPI2 in HTR8-S/Vneo cells. (a) The suspected binding of mutated 3′-UTR region of TFPI2 mRNA and hsa-miR-195 with the wild type is shown. (b) HEK-293T and HTR8-S/Vneo cells were transfected with indicated luciferase reporters. Relative luciferase activities were measured after incubation for 48 hr, (b and c). (d) HTR8-S/Vneo cells were transfected with negative control or miR-195 mimics for 48 hr. qRT-PCR was used to analyze the expressions of miR-195. (e) HTR8-S/Vneo cells were transfected with miR-195 mimics or negative control for 48 hr. Western blotting was used to measure the protein expressions of TFPI2. *n* = 3. Data were presented as mean ± SD.  ^*∗∗*^*p* < 0.01 and  ^*∗∗∗*^*p* < 0.001. One-way analysis of variance (ANOVA) followed Tukey's multiple comparisons test. mRNA, messenger ribonucleic acid; miR-195, microRNA-195; qRT-PCR, quantitative reverse transcription polymerase chain reaction; UTR, untranslated region.

We further examined the knockdown and overexpression of TFPI2 on cell proliferation and migration of HTR8-S/Vneo cells. Knockdown and overexpression of TFPI2 were successfully achieved verified by checking the mRNA expression of TFPI2 (Figures S2A and S2D). It was further found that knockdown of TFPI2 promoted the proliferation and migration of HTR8-S/Vneo cells (Figures S2B and 2C), while overexpression of TFPI2 did not affect the proliferation (Figure S2E) but inhibited the cell migration (Figure S2F). These results demonstrated a negative correlation between TFPI2 expression and cell proliferation and migration. To evaluate whether miR-195 targeted TFPI2, we analyzed the binding site of hsa-miR-195 with the wild-type (WT) 3'-UTR region of TFPI2 mRNA (Figure 5(a)). By cotransfecting HTR8-S/Vneo and HEK-293T cells with luciferase reporters containing WT or mutant TFPI2 3'-UTR, as well as miR-195 mimics and negative control, we found that luciferase activities of WT TFPI2-3'-UTR reporter were significantly reduced with miR-195 mimics in both HEK293T and HTR8-S/Vneo cells but showed no change in mutant TFPI2-3'-UTR reporter activities (Figures 5(b) and 5(c)). We next transfected HTR8-S/Vneo cells with miR-195 mimics or negative control (Figure 5(d)), and found that TFPI2 protein level was decreased in cells with miR-195 mimics transfection (Figures 5(e) and 5(f)), suggesting that TFPI2 was a target gene of miR-195.

### 3.6. EVs with miR-195 Overexpression Boosted HTR8-S/Vneo Cell Proliferation and Migration

**Figure 6:**
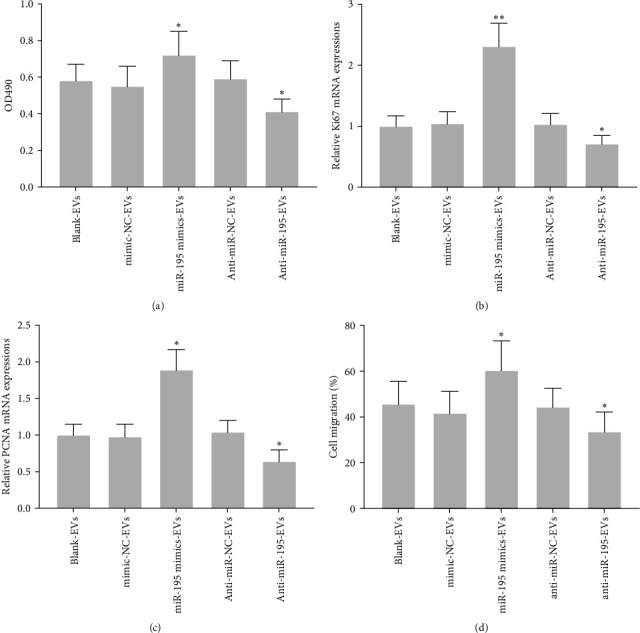
Elevated extracellular vesicle (EV)-derived miR-195 from human umbilical cord mesenchymal stem cell (hUC-MSCs) boosted HTR8-S/Vneo cell proliferation and migration. (a) Detection of HTR8-S/Vneo cell proliferation in each group by MTT assay. qRT-PCR was used to analyze the mRNA expressions of Ki67 (b) and PCNA (c). Relative expressions were normalized to blank-EVs. (d) Detection of HTR8-S/Vneo cell migration ability by transwell assay and the ratio of cell migration. *n* = 3. Data were presented as mean ± SD.  ^*∗*^*p* < 0.05 and  ^*∗∗*^*p* < 0.01 compared to blank-EVs. One-way analysis of variance (ANOVA) followed Tukey's multiple comparisons test. Ki67, nucleus related antigen 67; mRNA, messenger ribonucleic acid; miR-195, microRNA-195; MTT, 3-(4,5-dimethylthiazol-2-yl)-2,5-diphenyltetrazolium bromide; PCNA, proliferating cell nuclear antigen; qRT-PCR, quantitative reverse transcription polymerase chain reaction.

To investigate whether miR-195 overexpressed EVs affected cell proliferation and migration, we cocultured HTR8-S/Vneo cells with EVs containing either miR-195 mimics (miR-195 mimics-EVs group) or anti-miR-195 (anti-miR-195-EVs group). MTT assay results showed that miR-195 mimics-EVs enhanced cell proliferation and the mRNA levels of Ki67 and PCNA, whereas anti-miR-195-EVs significantly inhibited cell proliferation (Figures 6(a) and 6(c)). In addition, miR-195 mimics-EVs also elevated cell migration, whereas anti-miR-195 -EVs decreased cell migration capability (Figure 6(d)). These results clearly demonstrate the potential of overexpression of miR-195 in the EVs to enhance the cell proliferation and migration in HTR8-S/Vneo cells.

## 4. Discussion

Body fluids contain EVs and other secreted proteins, which provide clinical information and improve diagnostic accuracy for treatment selection [22]. For example, human ribonuclease 7 protein, which is secreted from cancer cells, has been reported as a potential biomarker in hepatocellular carcinoma targeted therapy [23]. EVs are small lipid-enclosed particles released by multiple cell types containing components such as noncoding RNAs, DNA fragments, mRNAs, and proteins, and play a critical role in cross talk between cells [24]. Multiple studies have highlighted the utility of tissue-specific EVs in the diagnosis of preeclampsia [25, 26]. In presymptomatic women, the concentration of total exosomes and placenta-derived exosomes in maternal plasma was significantly higher than those observed in controls [27]. Placenta-derived exosomes have been reported as potential biomarkers of preeclampsia [28]. In the current study, we have discovered that EVs derived from hUC-MSCs are potential liquid biopsy-based biomarkers for preeclampsia development.

Wang et al. [5, 10] reported the connection between multiple myeloma (MM) cells and bone marrow stromal cells (BMSCs). They found that exosomes derived from BMSCs regulated the activation of c-Jun, p53, p38, and Akt signaling, which could induce drug resistance in MM cells and prolong their survival. Zhang et al. [29] reported that hUC-MSC-derived exosomes played a critical role in the activation of the Wnt signaling pathway, leading to cell proliferation and wound re-epithelialization. In addition, microvesicles derived from human liver stem cells can promote hepatic regeneration through transferring microRNAs and proteins [30]. Consistent with previous studies, we found that hUC-MSC-derived EVs elevated HTR8-S/Vneo cell proliferation and migration abilities in vitro.

To investigate the role of exosomal miR-195 in the development of preeclampsia trophoblast cells, we transfected miR-195 into hUC-MSC-derived EVs and HTR8-S/Vneo cells. We observed that EV-derived miR-195 significantly enhanced HTR8-S/Vneo cell proliferation and migration. Furthermore, we found that EVs upregulated miR-195 expression in HTR8-S/Vneo cells, possibly due to protection from degradation. Luciferase reporter assay data indicated that miR-195 binds to the 3'UTR region of the TFPI2 gene. After coculturing hUC-MSC-derived EVs with HTR8-S/Vneo cells, TFPI2 expression was significantly decreased, suggesting that miR-195 could regulate trophoblast cell proliferation and migration by targeting TFPI2. A previous study reported that TFPI2 is a target of miR-195, involved in the regulation of glioblastoma cell proliferation and apoptosis [11]. In our study, we found a negative correlation between TFPI2 and miR-195 expression in preeclampsia patients and showed TFPI2 to be a target gene of miR-195. Our findings are in line with previous work that studied the role of exosomal miR-195 through an in vitro hypoxia model [14], but our study has more clinical relevance, as we investigated the correlation between TFPI2 and miR-195 in the placenta.

TFPI2 belongs to the Kunitz-type serine proteinase inhibitor family and functions as a tumor suppressor gene in multiple cancer types due to its ability to inhibit tumor cell growth, metastasis, and invasion [31, 32]. TFPI2 inhibits various serine proteases, such as plasmin, trypsin, VIIa/tissue factor, chymotrypsin, and plasma kallikrein [33]. Many studies have demonstrated that TFPI2 plays a role in the development of preeclampsia. For example, Zhou et al. [34] reported that upregulation of TFPI2 resulted in decreased cell proliferation and invasion, and induced cell apoptosis. TFPI2 was also found to be involved in the regulation of methylation status on the promoter region in preeclampsia placentas [35]. In the current study, we discovered that TFPI2 mRNA levels were significantly upregulated in preeclampsia tissues and were negatively correlated with miR-195 expression levels. Further luciferase assay indicated that miR-195 could bind to the 3'-UTR region of WT TFPI2 mRNA but not to the region of mutant TFPI2 construct. Additionally, miR-195 mimics significantly decreased TFPI2 protein expression, further suggesting that TFPI2 is a potential target of miR-195.

## 5. Conclusion

Our study demonstrates that the upregulation of miR-195 in EVs derived from hUC-MSCs promotes HTR8-S/Vneo cell proliferation and migration by targeting TFPI2. Our findings provide a potential therapeutic target to inhibit human extravillous cytotrophoblast-derived transformed cell proliferation and migration.

## Figures and Tables

**Table 1:** Clinical characteristics of preeclampsia and normal pregnancies.

	Normal *n* = 40	PE *n* = 34	*P* value
Maternal age (years)	26.4 ± 3.7	27.6 ± 3.9	0.164
Body mass index (kg/m^2^)	25.1 ± 4.4	25.7 ± 5.1	0.085
Time of pregnancy before delivery (week)	39.1 ± 0.7	32.9 ± 1.6	0.021
Systolic blood pressure (mm Hg)	112.3 ± 3.5	163.1 ± 9.2	0.005
Diastolic blood pressure (mm Hg)	71.7 ± 3.1	108.2 ± 9.3	0.002
Proteinuria (g/d)	0.15 ± 0.06	1.39 ± 0.71	<0.001
Blood urea nitrogen (U/L)	3.76 ± 1.13	4.02 ± 1.25	0.132
Blood platelet (×10^9^)	176.22 ± 60.31	170.36 ± 64.09	0.213
Body weight of infant (g)	3316 ± 215	2208 ± 623	0.012

*Note*. The data presented are mean ± SD. The comparison of data between the two groups was done by unpaired Student's *t*-test.

## Data Availability

The raw data supporting the conclusions of this article will be made available by the authors, without undue reservation.

## Supplementary Materials

Supplementary Materials Figure S1: Detection of Alix expression by western blot analysis in hUC-MSC-derived EVs. Figure S2: Overexpression of TFPI2 would inhibit proliferation and migration of HTR8-S/Vneo cells, but knockdown of TFPI2 would promote proliferation and migration of HTR8-S/Vneo cells.
