# Supplementary material for: Extracellular Vesicles Derived from Human Umbilical Cord Mesenchymal Stem Cells Promote Trophoblast Cell Proliferation and Migration by Targeting TFPI2 in Preeclampsia
Source: Stem Cells Int. 2023 Aug 1;2023:7927747. doi: 10.1155/2023/7927747 (PMC10409582; doi:10.1155/2023/7927747)
Supplement: Supplementary Materials — Figure S1: Detection of Alix expression by western blot analysis in hUC-MSC-derived EVs. Figure S2: Overexpression of TFPI2 would inhibit proliferation and migration of HTR8-S/Vneo cells, but knockdown of TFPI2 would promote proliferation and migration of HTR8-S/Vneo cells. [file 7927747.f1.docx]

**Supplementary materials**


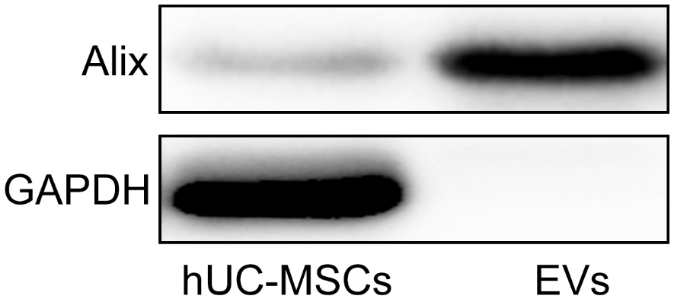


Figure S1. Detection of Alix expression by Western blot analysis in hUC-MSC-derived extracellular vesicles.


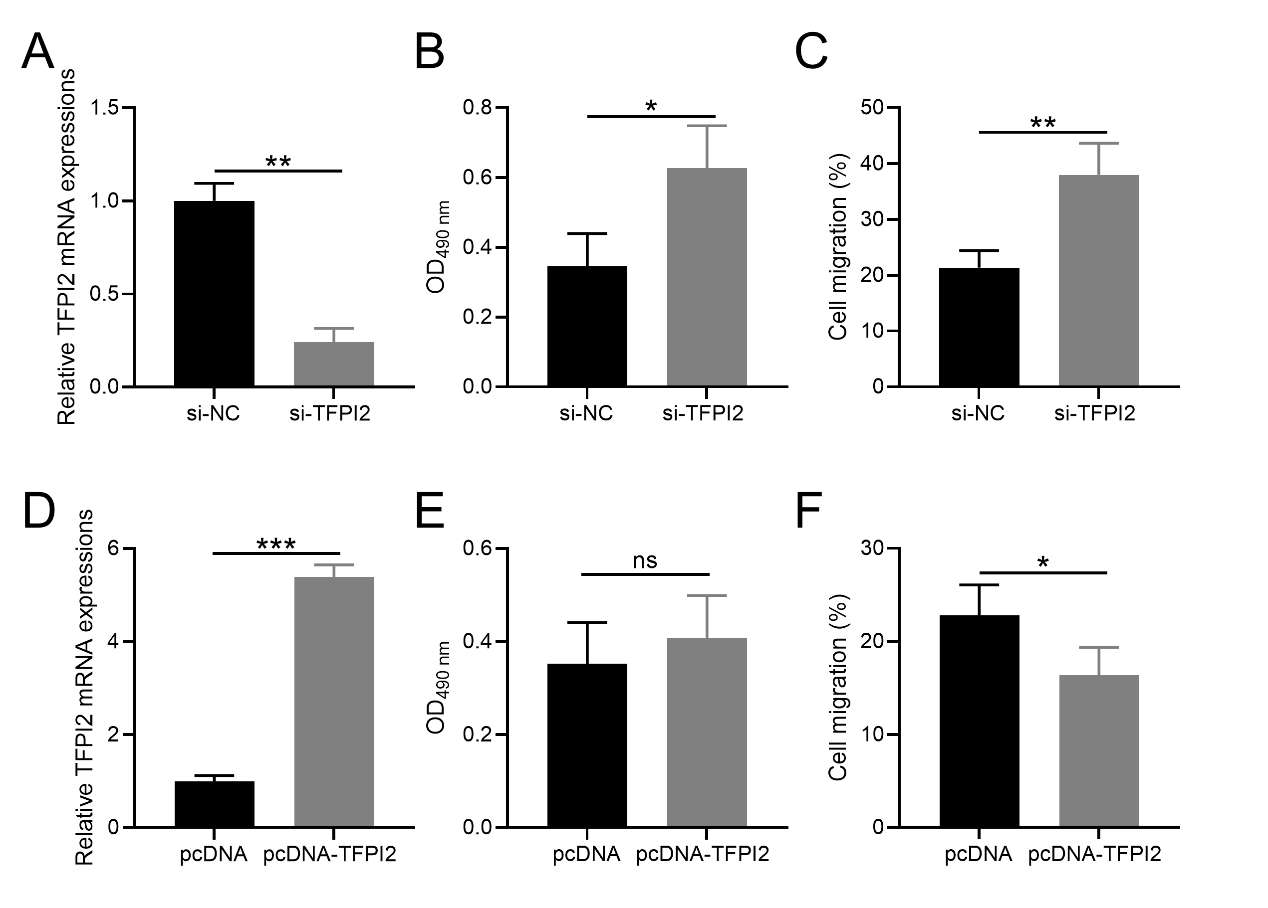


Figure S2. Overexpression of TFPI2 would inhibit proliferation and migration of HTR-8/SVneo cells but knockdown of TFPI2 would promote proliferation and migration of HTR-8/SVneo cells. qRT-PCR was used to confirm the efficiency of overexpression or knockdown of TFPI2 in HTR-8/SVneo cells (A and D). Detection of HTR8-S/Vneo cell proliferation in each group by MTT assay (B and E). Detection of HTR8-S/Vneo cell migration ability by Transwell assay and the ratio of cell migration (C and F). Data were presented as mean ± SD. * p < 0.05, ** p < 0.01, *** p < 0.001 and ns means no significance, Mann Whitney test.
